# Phenylpropenoic Acid Glucoside from Rooibos Protects Pancreatic Beta Cells against Cell Death Induced by Acute Injury

**DOI:** 10.1371/journal.pone.0157604

**Published:** 2016-06-14

**Authors:** Eddy Himpe, Daniel A. Cunha, Imane Song, Marco Bugliani, Piero Marchetti, Miriam Cnop, Luc Bouwens

**Affiliations:** 1 Cell Differentiation Lab, Diabetes Research Center, Vrije Universiteit Brussel (VUB), Brussels, Belgium; 2 ULB Center for Diabetes Research, Université Libre de Bruxelles (ULB), Brussels, Belgium; 3 Division of Endocrinology, Erasmus Hospital, Brussels, Belgium; 4 Department of Endocrinology and Metabolism, University of Pisa, Pisa, Italy; Universidad Miguel Hernández de Elche, SPAIN

## Abstract

**Objective:**

Previous studies demonstrated that a phenylpropenoic acid glucoside (PPAG) from rooibos (*Aspalathus linearis*) extract had anti-hyperglycemic activity and significant protective effects on the pancreatic beta cell mass in a chronic diet-induced diabetes model. The present study evaluated the cytoprotective effect of the phytochemical on beta cells exposed to acute cell stress.

**Methods:**

Synthetically prepared PPAG was administered orally in mice treated with a single dose of streptozotocin to acutely induce beta cell death and hyperglycemia. Its effect was assessed on beta cell mass, proliferation and apoptotic cell death. Its cytoprotective effect was also studied in vitro on INS-1E beta cells and on human pancreatic islet cells.

**Results:**

Treatment with the phytochemical PPAG protected beta cells during the first days after the insult against apoptotic cell death, as evidenced by TUNEL staining, and prevented loss of expression of anti-apoptotic protein BCL2 in vivo. In vitro, PPAG protected INS-1E beta cells from streptozotocin-induced apoptosis and necrosis in a BCL2-dependent and independent way, respectively, depending on glucose concentration. PPAG also protected human pancreatic islet cells against the cytotoxic action of the fatty acid palmitate.

**Conclusions:**

These findings show the potential use of PPAG as phytomedicine which protects the beta cell mass exposed to acute diabetogenic stress.

## 1. Introduction

Regulation of the blood glucose level after a meal depends on the pancreatic insulin-producing beta cells. High-caloric western diets rich in saturated fats and sugars lead to obesity and insulin resistance which increases the secretory demand on beta cells. As a result, beta cells are exposed to oxidative stress and endoplasmic reticulum (ER) stress which potentially impair their function and survival. However, beta cells need to compensate for the increasing insulin demands by raising insulin synthesis and secretion. Failure to compensate leads to a vicious circle of increased metabolic stress and decreased beta-cell number which underlies the pathogenesis and progression of type 2 diabetes [1]. Type 2 diabetes is a chronic metabolic disease with increasing prevalence worldwide. There is an urgent need to find new anti-diabetic drugs that not only decrease glycemia but also preserve beta cell mass and thereby would be disease-modifying [2]. There is also interest in the potential use of dietary supplements or nutraceuticals that promote preservation of the beta cell mass in pre-diabetic or at risk individuals [3].

Natural products play a dominant role in the discovery of leads for the development of drugs for the treatment of human diseases. Previous studies have attributed a glucose-lowering effect to a phytochemical substance from rooibos (*Aspalathus linearis*), namely phenylpropenoic acid glucoside (PPAG) [4]. We recently reported that oral PPAG administration to mice that were fed a high fat and fructose diet (mimicking an unhealthy western diet) prevented the mice from developing diabetes [5]. PPAG treatment in this chronic, long-term (12 weeks) experimental model increased beta cell mass by decreasing lipotoxic beta cell apoptosis. PPAG also has a hypoglycemic effect [4] and could thereby exert a beta cell protective effect by attenuating glucotoxicity.

The present study was designed to examine a possible direct beta–cytoprotective effect of acute oral treatment as opposed to chronic treatment with PPAG. Diabetes was induced in mice by a single high-dose streptozotocin (STZ) injection. We examined beta cell mass, proliferation and apoptotic cell death in vivo, and further studied the mechanism of cell death in vitro. We also examined whether PPAG protects human islet cells against a diabetogenic insult.

Our results show that PPAG protects pancreatic beta cells against the acute toxic effects of STZ, oxidative stress and glucotoxicity and has both anti-apoptotic and anti-necrotic effects.

## 2. Materials and Methods

### 2.1. Animals and experimental design

Animal procedures were approved by our institutional ethical committee of the Vrije Universiteit Brussel (permit number: LA1230277) and performed in accordance with the national guidelines and regulations.Approval was obtained for this specific study (12-277-1). Animals were housed in the university animal house according to the regulations of Belgian and EU legislation; food and water supply was given ad libitum. Animal pain and suffering was assessed as “class 3” by the ethical committee, requiring no special treatment.Male Balb/c mice, weighing approximately 25 g (n = 25), 9–11 weeks of age, were obtained from Charles River laboratories (Saint Germain Nuelles, France). Animals were divided over three groups: untreated controls, STZ-treated mice, STZ-treated mice receiving PPAG. PPAG dissolved in water was administered daily in a dose of 10 mg/kg body weight by oral gavage starting 48 hours prior to STZ injection until the end of the experiment. Animals were injected intraperitoneally with a single dose of STZ at 200 mg/kg body weight dissolved in freshly prepared 0.1 M citrate buffered saline (pH 4.5). Glycemia was measured at the tail end of the mice with a glucometer (GlucoMenLXPlus+, Menarini diagnostics, Zaventem, Belgium). Mice were euthanized by cervical dislocation on 30 hours or 11 days post-STZ injection.

### 2.2. PPAG

(Z)-2-(β-D-glucopyranosyloxy)-3-phenylpropenoic acid 1 (PPAG), a water-soluble enolic glucoside of phenylpyruvic acid [6], was prepared synthetically as described by Marais et al. [7] with a purity >99% based on HPLC. It was kindly supplied by MC2 Biotek Group (Horsholm, Denmark); development of PPAG as an anti-type 2 diabetes drug is pursued by MC2 Biotek under the name RX-1.

### 2.3. Beta cell mass

Beta cell mass was measured by morphometry in paraffin sections of the pancreas and calculated by multiplying the relative insulin-immunoreactive area by the pancreatic weight as previously described [8]. At least 100 mm^2^ of pancreatic tissue was measured per animal.

### 2.4. Apoptosis and proliferation in vivo

Beta cell proliferation was measured by Ki67 (Novocastra Laboratories, Newcastle, UK) immunostaining in paraffin sections. To measure apoptosis in tissue sections, TUNEL staining was performed using the cell death detection kit from Roche (Brussels, Belgium). Ki67 or TUNEL staining were combined with insulin immunostaining [5].

### 2.5. BCL2 expression in vivo

B-cell lymphoma 2 (BCL2) expression was determined in pancreatic islets by measuring the optical density in BCL2 stained pancreatic sections using Image J software (NIH, Maryland, USA) that was calibrated using the Rodbard method. Background was subtracted from at least ten representative pancreatic islets from each mouse. BCL2 staining was performed using the anti-BCL2 antibody from Upstate Biotechnology (04–436, Temecula, CA) and visualized with DAB staining.

### 2.6. Phospho-H2AX expression in vivo

DNA damage in beta cells was determined by immunostaining on paraffin sections of phosphorylated histone H2AX (Cell Signaling, Beverly, MA, USA). Phospho-H2AX staining was combined with insulin immunostaining.

### 2.7. INS-1E cells

The rat insulin-producing INS-1E cell line (a kind gift from Dr. C. Wollheim, Centre Medical Universitaire, Geneva, Switzerland) was cultured in RPMI 1640 (with 2 mM GlutaMAX-I) containing 5% FBS [9] and used at passages 60–71. PPAG was diluted in water and used at a concentration of 30 μM [5]. INS-1E cells were pre-incubated with PPAG for 16 hours, then exposed to 1 mM STZ for 1 hour and further cultured for the indicated times. INS-1E cells were exposed to hydrogen peroxide (30 μM) in RPMI for 30 minutes, after which medium was replaced to medium containing PPAG for another 24 hours.

### 2.8. Human islets

Human islets were isolated by collagenase digestion and density gradient purification from 4 donors (age 65 ± 9 years, BMI 25 ± 3 kg/m^2^, 3 male and 1 female, cause of death in all cases was cerebral hemorrhage) [10]. Human islets were isolated from the pancreas of non diabetic multi organ donors with the approval of the Ethics Committee of the University of Pisa. Pancreata were collected from brain-dead organ donors after informed consent was obtained in writing form from family members. The islets were cultured as described [11]. The percentage of beta cells, examined by insulin immunofluorescence [12], was 44 ± 6%. Human islets were exposed to 0.5 mM palmitate as described [11] with or without 30 μM PPAG, and cell death was assessed after 3 days [11].

### 2.9. Assessment of beta cell death in vitro

Apoptotic and necrotic INS-1E cells were counted under a fluorescence microscope after staining with the DNA-binding dyes propidium iodide (5 μg/mL) and Hoechst 33342 (10 μg/mL) [13]. Apoptosis was confirmed by additional methods, including caspase 3 and -9 cleavage (see below).

### 2.10. Western blot

Western blots were performed using 20 μg whole cell extract protein as described [14]. The primary antibodies were BCL2 (1/10000), cleaved caspase 3 (1/1000), cleaved caspase 9 (1/1000), α-tubulin (1/5000) from Sigma-Aldrich (Diegem, Belgium). Horseradish peroxidase-labeled goat anti-rabbit (1/5000, Sigma-Aldrich) or goat anti-mouse (1/10000, Pierce, Rockford, IL, USA) antibodies were used as secondary antibodies. Protein signal was visualized using chemiluminescence Supersignal (Pierce) and quantified using Scion Image (Scion Corporation, Frederick, MD, USA).

### 2.11. DCF fluorescence

Oxidative stress was measured using the fluorescent probe 2, 7-dichlorofluorescein diacetate (DCF) (Sigma-Aldrich) in INS-1E cells seeded in black 96-well plates. After treatment, cells were loaded with 10 μM DCF for 30 minutes at 37°C and washed. DCF fluorescence was quantified in Victor 2 reader (Perkin Elmer, Germany). Cells were then lysed and total protein measured. Data are expressed as DCF fluorescence corrected by total protein.

### 2.12. Statistics

In the in vivo experiments, there were six groups: Mice treated with STZ and sacrificed at 30 hours after STZ (n = 7) or after 11 days (n = 3), mice treated with PPAG and STZ sacrificed at 30 hours after STZ (n = 8) or after 11 days (n = 4), and untreated mice sacrificed at 30 hours after STZ (n = 10) or after 11 days (n **=** 5). Statistical analyses of the in vivo and in vitro experiments were carried out by 2-way ANOVA followed by Bonferroni post tests using GraphPad Prism 5. For the in vitro experiments, comparisons were made by two-sided paired t test with Bonferroni correction for multiple comparisons where appropriate. Data are presented as means ± SEM. A p-value of **<**0.05 was taken as statistically significant.

## 3. Results

### 3.1. Blood glucose and beta cell mass in streptozotocin-induced diabetic mice

**Fig 1:**
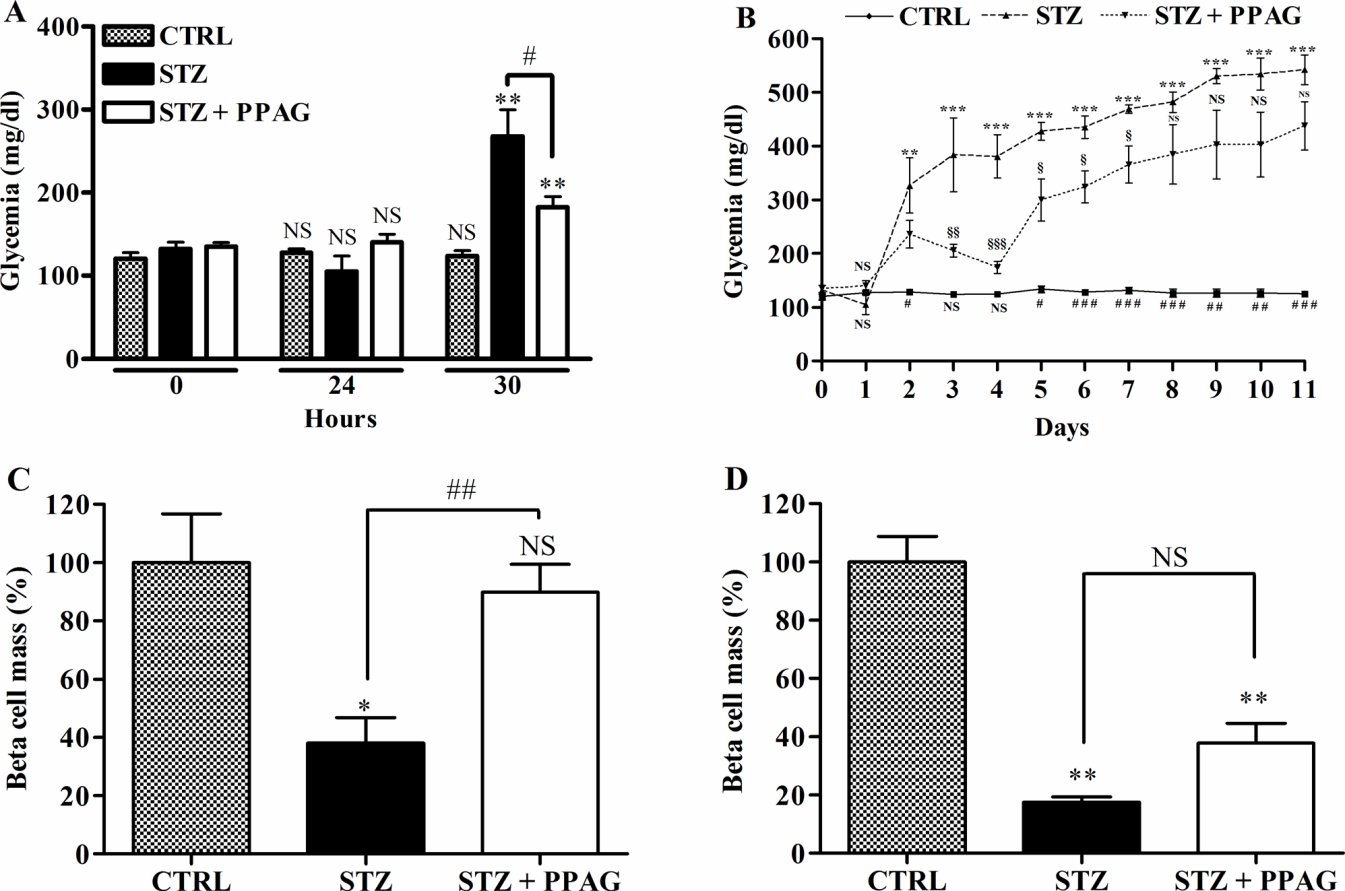
Effect of STZ and PPAG on blood glucose levels and beta cell mass in vivo. Shaded bars: untreated controls, CTRL (n = 10); filled bars: STZ-treated animals, STZ (n = 7); open bars: STZ-treated animals that were treated with PPAG, STZ+PPAG (n = 8). (A) Non-fasting blood glucose levels immediately before and 30 hours after a single injection of STZ. (B) Daily follow up of non-fasting blood glucose from immediately before a single injection of STZ until 11 days. Values are mean ± SEM. ANOVA with Bonferroni post test was used to determine the significance between groups on each day. **p<0.01; ***p<0.001 between control and STZ group. #p<0.05; ## p<0.01; ###p< 0.001 between control and STZ + PPAG group. § p<0.05; §§ p<0.01; §§§ p< 0.001 between STZ and STZ + PPAG group. (C) Percentage of beta cell mass normalized to the control 30 hours (C) or 11 days (D) after STZ injection. Data shown are means ± SEM. **p<0.01; #p<0.05; NS: not significant.

The aim of this study was to examine whether the phytochemical PPAG has a beta cell protective effect in a model of acute damage for which we used a high dose of STZ. Animals were hyperglycemic (>250 mg/dl) at 30 hours after STZ injection and their glycemia further rose to nearly 400 mg/dl at 4 days and 500 mg/dl at 11 days (Fig 1A and 1B). Animals that were pretreated with PPAG developed hyperglycemia 30 hours after STZ but values remained below 200 mg/dl which was significantly lower than in STZ-only mice. After 4 days their blood glucose improved to near normal levels. However, at 11 days the PPAG treated animals also had become overtly hyperglycemic.

We quantified beta cell mass at 30 hours and 11 days post-STZ (Fig 1C). At 30 hours, the beta cell mass of STZ-treated animals was significantly decreased by about 60% whereas the beta cell mass of PPAG treated animals was unchanged. At 11 days (Fig 1D), however, the beta cell mass in the PPAG group was reduced by about 60% compared to an 80% decrease in the STZ group.

Thus, treatment with the phytochemical PPAG delayed the onset of hyperglycemia and protected beta cells against the acute cytotoxic effect of high-dose STZ but not against its long-term toxic effect.

### 3.2. Beta cell apoptosis, proliferation and BCL2 expression in streptozotocin-induced diabetic mice

**Fig 2:**
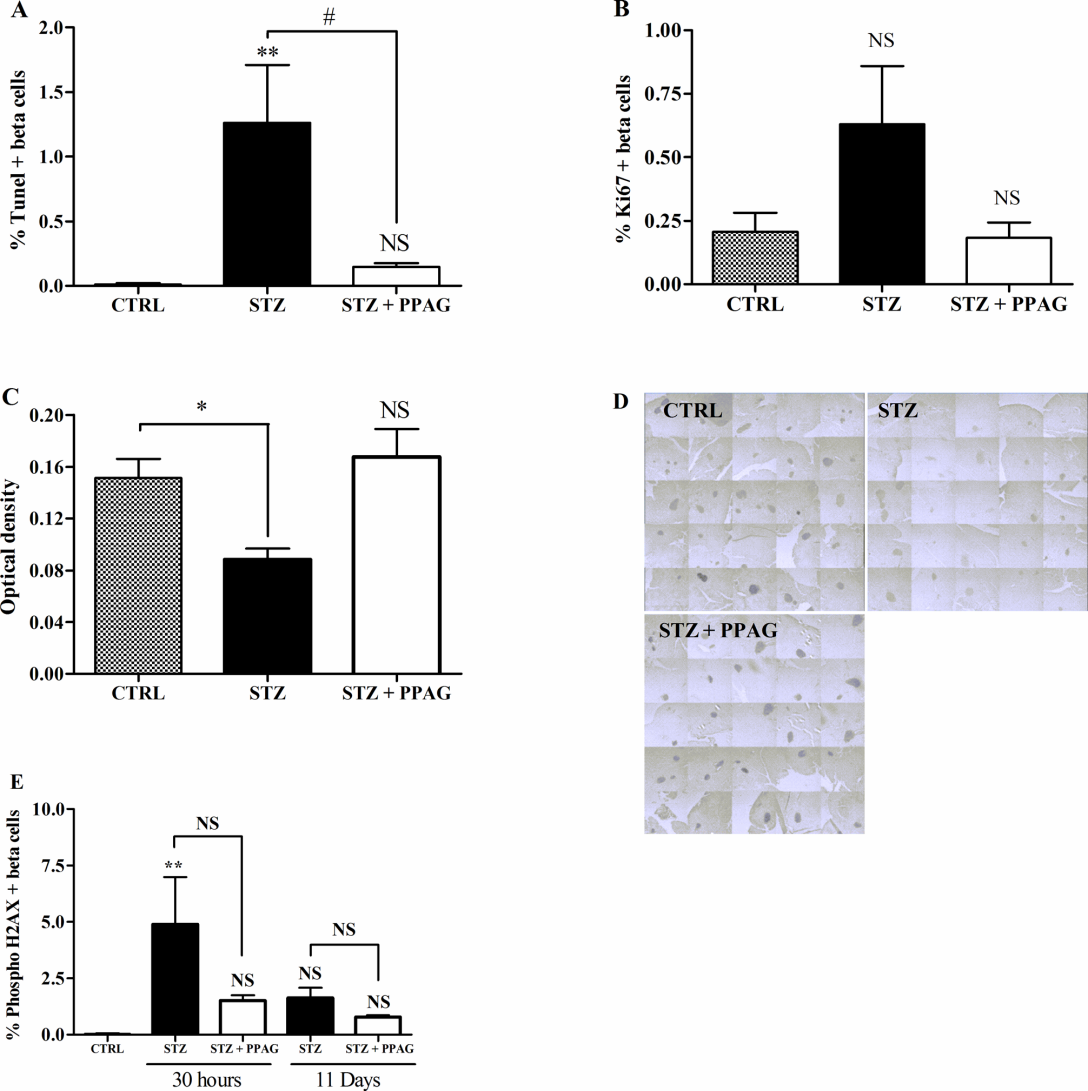
Effect of STZ and PPAG on beta cell apoptosis/proliferation and the expression of BCL2 and phospho H2AX. Shaded bars: untreated controls, CTRL (n = 5); filled bars: STZ-treated animals, STZ (n = 4); open bars: STZ-treated animals that were treated with PPAG, STZ+PPAG (n = 4). (A) Percentage of TUNEL stained cells in the insulin-positive cell population in pancreatic tissue sections. (B) Percentage of Ki67 stained cells in the insulin-positive cell population in pancreatic tissue sections. Values are mean ± SEM. Significance compared to the control was determined by one-way ANOVA with Dunnett post-test. **P<0.01; NS (not significant). For the effect of PPAG on STZ induced apoptosis a one-sample t-test was used. #P<0.05. (C) Densitometric quantification of BCL2 expression in the islets. Values are mean ± SEM. Significance compared with the control was determined using an one-way ANOVA with a Dunnett post-test. **P<0.01; NS (not significant). (D) Representative images of the BCL2 staining in pancreatic islets, 30 hours post-STZ. (E) Beta cell DNA damage/repair. Shaded bars: untreated controls, CTRL (n = 5); filled bars: STZ-treated animals, STZ (n = 4 for 30 hours and n = 3 for 11 days); open bars: STZ-treated animals that were treated with PPAG, STZ+PPAG (n = 4 for 30 hours and n = 4 for 11 days). No positive cells were detected in control animals. Results from STZ and STZ+PPAG were not statistically different (p≥0.05). **p<0.01 between control group and STZ.

To ascertain that PPAG preserved beta cell mass by exerting a beta-cytoprotective effect, we quantified the number of beta cells that were positive for TUNEL staining, a marker of cells undergoing apoptosis. STZ induced a significant increase in apoptotic beta cells and PPAG nearly completely abolished this effect at 30 hours after STZ (Fig 2A). At later time points, the number of apoptotic beta cells was negligible and similar to untreated control animals. We also examined the number of insulin-positive cells expressing the Ki-67 marker for proliferation at 30 hours post-STZ. There were no significant differences in the number of proliferating beta cells between groups (Fig 2B).

Next we examined whether STZ-induced beta cell death and the protective effect of PPAG were correlated with expression levels of the anti-apoptotic protein BCL2. In a recent study we found that PPAG protects against fatty acid-induced beta cell death by preventing BCL2 degradation [5]. We therefore semi-quantitatively assessed BCL2 expression in immunostained pancreas sections. Islets of Langerhans stained more intensely in the PPAG and untreated control group than in the STZ group (Fig 2C and 2D). This indicates that PPAG treatment is associated with preserved expression of anti-apoptotic BCL2 protein that prevents the triggering of the apoptotic cell death process.

### 3.3. Effect on DNA damage

STZ is known to cause DNA damage by its alkylating activity and by acting as a NO-donor, thereby inducing DNA repair. We assessed this by immunohistochemical staining of phosphorylated histone H2AX, a marker of DNA damage/repair, in insulin-positive beta cells at 30 hours and 11 days post-STZ. There was a non significant trend towards higher number of positive beta cells at 30 hours and 11 days in the STZ group compared to STZ + PPAG group. The percent H2AX-positive beta cells varied between 1 and 5% in STZ-treated animals and was not significantly different from PPAG-treated and untreated animals at both time points (Fig 2E). H2AX-positive cells were practically undetectable in untreated controls. These observations indicate that DNA-damage was present throughout the experimental period and was unaffected by PPAG.

### 3.4. Protective effect of PPAG in vitro

**Fig 3:**
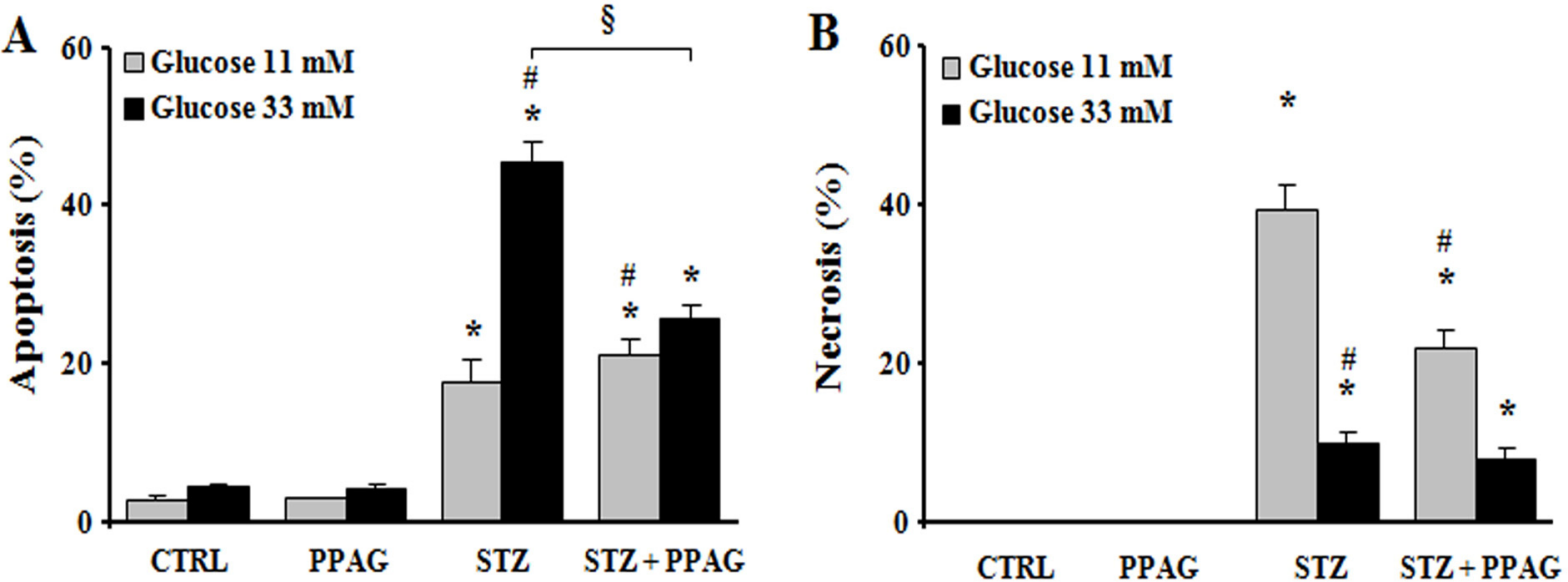
In vitro beta cell protection from STZ by PPAG. INS-1E cells were pre-treated with 30 μM PPAG for 16 hours, exposed to 1 mM streptozotocin for 1 hour and then cultured for 23 hours in medium containing 11 or 33 mM glucose with or without PPAG (n = 3). The percentage of apoptotic (A) and necrotic cells (B) was determined following staining with the nuclear dyes propidium iodide and Hoechst 33342. A minimum of 500 cells was counted for each condition. Percentage of apoptotic (A) and necrotic cells (B). *p<0.05 against control (CTRL). #p<0.05 against STZ-treated cells in 11 mM glucose. §p<0.05 as indicated.

The cytoprotective effect of PPAG was further examined in the rat beta cell line INS-1E in vitro. For this purpose, INS-1E cells were exposed for 1 hour to 1 mM STZ which induces 50–60% cell death. This is comparable to the extent of cell loss observed in vivo 30 hours post-STZ. To further mimic the in vivo conditions, after the STZ injury the INS-1E cells were exposed to an elevated glucose concentration (33 mM, compared to the normal in vitro glucose concentration of 11 mM). The STZ plus high glucose insult induced 40% apoptosis and 10% necrosis (Fig 3). PPAG added to the culture medium before and after STZ halved apoptotic cell death (Fig 3A). At 11 mM glucose STZ induced 20% apoptotic and 40% necrotic cell death. In these conditions PPAG did not inhibit STZ-induced apoptosis but it significantly reduced the number of necrotic cells (Fig 3B).

**Fig 4:**
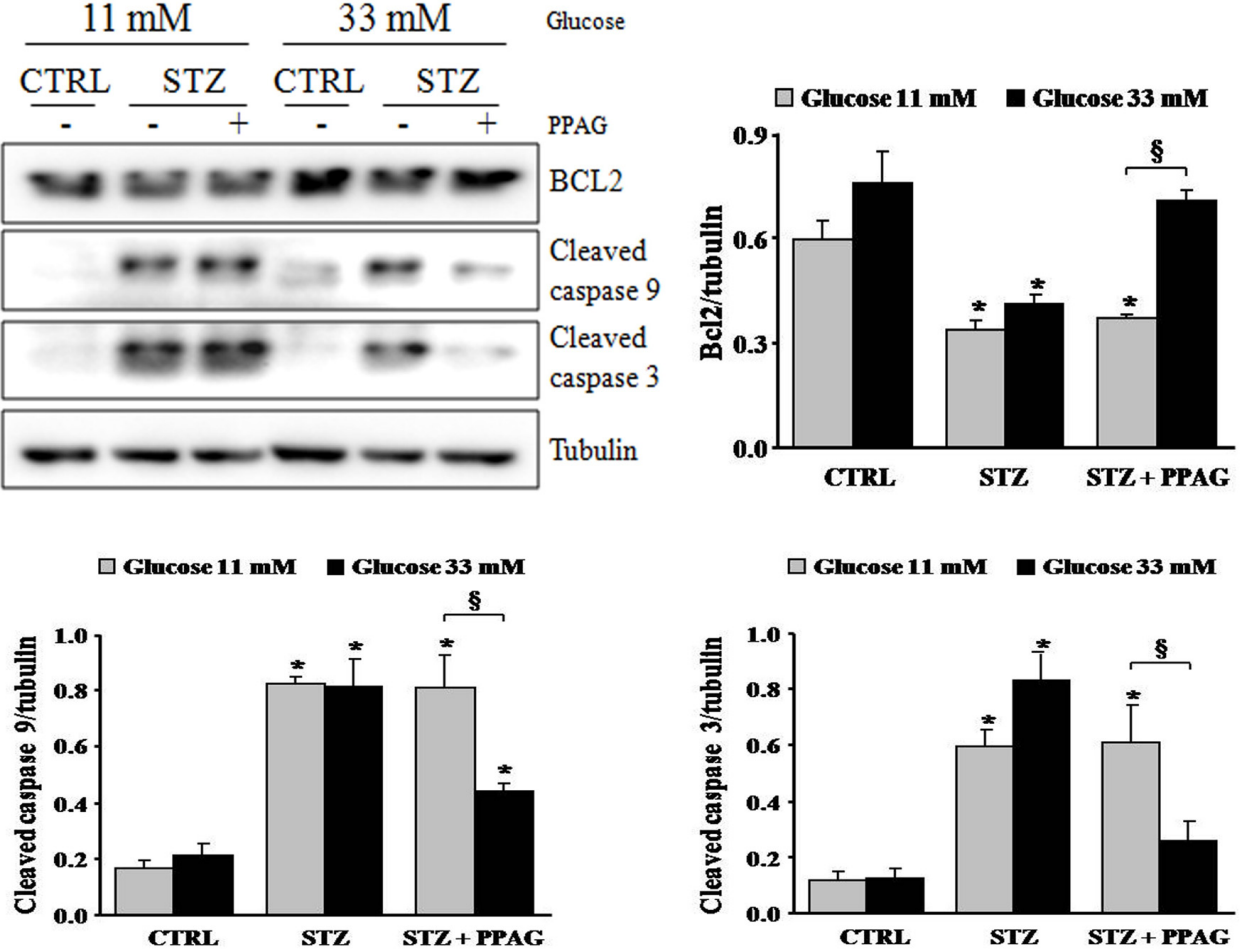
Expression of caspases and BCL2. Western blot and densitometry for BCL2 and cleaved caspase 9 and 3 in INS-1E cells pre-treated with 30 μM PPAG for 16 hours, exposed to 1 mM STZ for 1 hour and then cultured for 7 hours in medium containing 11 or 33 mM glucose with or without PPAG (n = 3).*p<0.05 against control (CTRL). §p<0.05 as indicated.

The apoptotic cell death process was further examined by Western blot (Fig 4). STZ induced cleavage of caspase-9 and caspase-3 in INS-1E cells, which is a hallmark of apoptotic cell death. STZ decreased expression of the anti-apoptotic protein BCL2. Interestingly, PPAG inhibited caspase activation in beta cells cultured at elevated glucose following STZ, and it did not affect caspase cleavage at normal glucose level. In keeping with these data, PPAG restored BCL2 expression levels at elevated but not at normal glucose level.

In conclusion, in elevated glucose conditions STZ induced predominately apoptotic beta cell death and PPAG was beta-protective in a BCL2-dependent manner. At lower glucose concentrations, STZ favored necrotic cell death. PPAG also protected against this beta-cytotoxic effect, but in a BCL2-independent manner.

**Fig 5:**
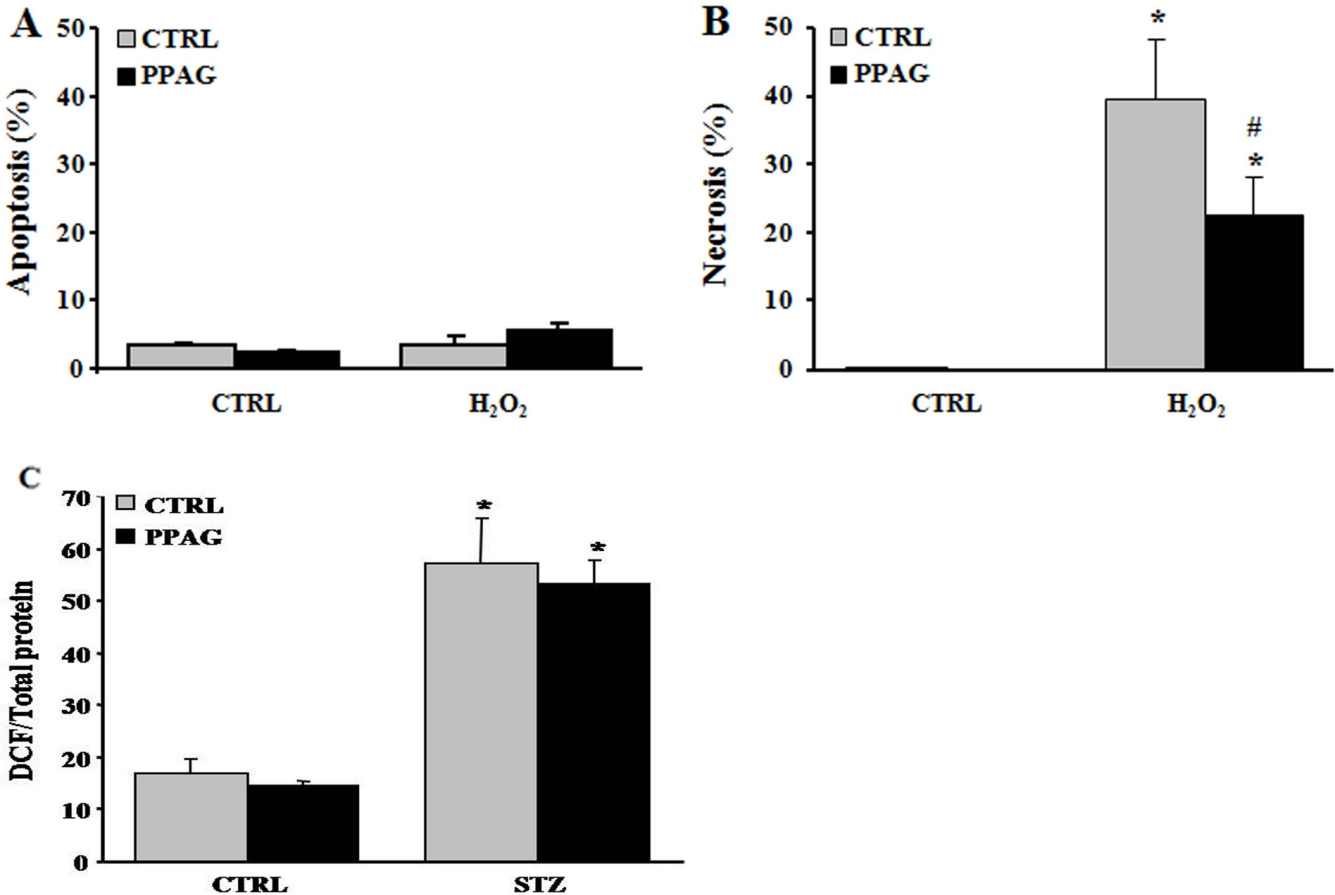
In vitro beta cell protection from hydrogen peroxide by PPAG and the effect of PPAG on oxidative stress. Cell death in INS-1E cells pre-treated with 30 μM PPAG for 16 hours, exposed to 30 μM hydrogen peroxide (H_2_O_2_) for 30 min, and then cultured for 24 hours in control medium with or without PPAG (n = 4). The percentage of apoptotic (A) and necrotic cells (B) was determined following staining with the nuclear dyes propidium iodide and Hoechst 33342. A minimum of 500 cells was counted for each condition. Percentage of apoptotic (A) and necrotic cells (B).*p<0.05 against untreated cells. #p<0.05 against H_2_O_2_-treated cells. (C) INS-1E cells were pre-treated for 16 hours with 30 μM PPAG, exposed to 1 mM STZ for 1 hour, and further cultured for 2 hours in the presence of absence of PPAG (n = 3). Oxidative stress was measured by dichlorofluorescein (DCF) oxidation. *p<0.05 against untreated cells.

We previously reported that PPAG protects against apoptotic beta cell death induced by fatty acids and ER stress by preserving BCL2 expression while it had no effect on the expression of pro-apoptotic signals [5]. To further explore the anti-necrotic effect of PPAG discovered in the present study, we examined another necrosis-inducing agent, namely hydrogen peroxide. Twenty-four hours after a 30-minute exposure to hydrogen peroxide, 40% of INS-1E cells underwent necrotic cell death, and no apoptosis was induced (Fig 5A). PPAG significantly decreased this necrotic cell death (Fig 5B).

STZ is known to exert its beta-cytotoxic effect amongst others by the formation of superoxide radicals that increase oxidative stress to a cytotoxic level [20]. We therefore examined whether PPAG reduces oxidative stress by assessing oxidation of dichlorofluorescein (DCF), an indicator for reactive oxygen species. PPAG had no effect on DCF fluorescence compared to cells treated with STZ alone (Fig 5C). Therefore, it is concluded that PPAG does not act as an antioxidant but rather exerts downstream protection against oxidative stress-induced cell death.

### 3.5. Protection of human islet cells

**Fig 6:**
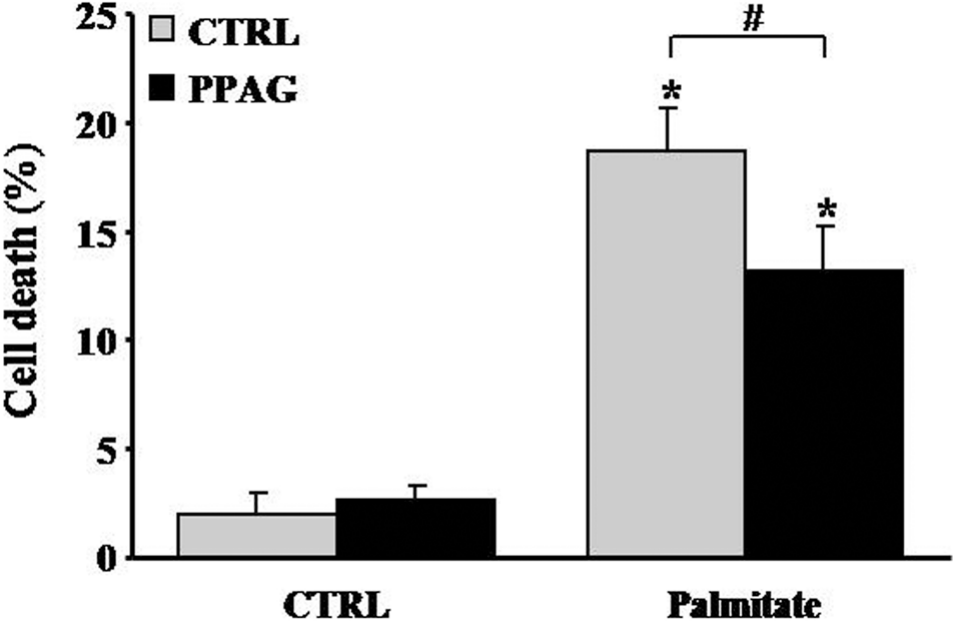
Effect of PPAG on lipotoxic cell death in human islet cells. Human islets were treated with 0.5 mM palmitate and 30 μM PPAG or vehicle for 3 days (n = 4). The percentage of apoptotic (A) and necrotic cells (B) was determined following staining with the nuclear dyes propidium iodide and Hoechst 33342. A minimum of 500 cells was counted for each condition. *p<0.05 against untreated cells, #p< 0.05 as indicated.

Our previous [5] and current findings convincingly demonstrate that PPAG is cytoprotective in mouse and rat beta cells. We next asked whether this effect in rodent cells can be reproduced in human cells, and examined whether PPAG protects human islets against a diabetogenic insult. Since human beta cells are resistant to the cytotoxic action of STZ [15] we opted for a lipotoxic injury induced by palmitate [5]. PPAG significantly reduced palmitate cytotoxicity in human islets (Fig 6), confirming that the phytochemical is also protective for human beta cells.

## 4. Discussion

The aim of this study was to assess the beta-cytoprotective effect of the rooibos phytochemical PPAG in a setting of acute damage. The single STZ injection model used in this study provides a cost-effective, time-saving, convenient platform for drug screening related to diabetic glucotoxicity [16]. The high STZ-dose used for this purpose in vivo induces rapidly and irreversibly massive beta cell loss and hyperglycemia [17]. In the short-term (30 hours), PPAG pretreatment nearly completely prevented STZ-induced beta cell apoptosis assessed by TUNEL staining and it preserved beta cell mass. This short time period following the STZ insult is very interesting as the fraction of apoptotic beta cells was between 1 and 2%. This is ten-fold higher than in chronic insult models where the fraction of apoptotic beta cells is so low that it becomes difficult to quantify [5]. In our previous study [5] we showed that daily PPAG administration (same dose as in the present study) does not affect the following parameters in mice: glycemia, plasma insulin, body weight, food and fluid intake, beta cell mass, proliferation and apoptosis, and Bcl2 expression. PPAG treatment also maintained near-normal glycemia for a period of 4 days after the insult. The beta cell mass did not differ significantly during the initial glucose increase at 30 hours (PPAG + STZ). This could be explained by a functional effect of STZ on beta cell insulin secretion. STZ also targets mitochondrial DNA thereby impairing the signaling function of beta cell mitochondrial metabolism which may affect glucose-induced insulin secretion.The beta-cytoprotective effect was accompanied by the preservation of a normal expression level of the anti-apoptotic protein BCL2 in pancreatic islets in vivo. We previously reported that PPAG-treated beta cells maintain a higher level of BCL2 expression in conditions of chronic lipotoxicity and ER stress-induced apoptosis [5]. In the longer term, between 4 and 11 days after the insult, the PPAG-treated animals became hyperglycemic and their beta cell mass was significantly reduced. This eventual beta cell demise could be explained by irreparable DNA-damage inflicted by STZ, a known genotoxic agent.

The protection of PPAG against the acute cytotoxicity of STZ was further investigated in an in vitro model using the INS-1E beta cell line, which has often been used in functional studies including screens of bioactive compounds [18]. PPAG pretreatment of INS-1E cells reduced STZ-induced cell death by 30–40%. Apoptosis, as a result of decreased BCL2 expression and caspase activation, was the prevalent mode of cell death in STZ-injured cells exposed to high glucose. Necrosis prevailed as the mode of cell death at normal glucose concentration. Under this condition, PPAG did not affect BCL2 expression or caspase activation, but it was also beta cell protective. In vivo, glucotoxicity is characterized by apoptotic beta cell death and this is considered to contribute to the development and progression of type 2 diabetes [19]. The observed protective effect of PPAG may thus be relevant to glucotoxicity as it occurs in type 2 diabetes. It is interesting that PPAG also protected INS-1E cells against necrotic cell death induced by hydrogen peroxide, an inducer of oxidative stress.

STZ is known to execute its cytotoxicity in the beta cell primarily as a result of its DNA damaging effect. STZ-induced beta cell death involves both apoptosis and necrosis [20–23] or a regulated form of necrotic cell death [24]. The methylnitrosurea group of STZ alkylates DNA and liberates NO, both causing DNA damage which in turn induces activation of poly-ADP-ribosylation involved in DNA repair. This leads to depletion of cellular NAD^+^ and as a consequence also of cellular ATP stores. ATP dephosphorylation supplies a substrate for xanthine oxidase resulting in the formation of superoxide radicals and thus increasing oxidative stress. Rodent beta cells are vulnerable to oxidative stress due to their low expression of antioxidant enzymes [25,26]. Marked ATP depletion prevents apoptosis since apoptosome formation is an energy-dependent process while lower grades of ATP depletion may lead to apoptotic cell death [27]. Likewise, hydrogen peroxide can induce cell death by apoptosis or necrosis depending on the severity of the oxidative stress, with marked ATP depletion switching the mode of cell death from apoptosis to necrosis [28].

The dichlorofluorescein oxidation assay confirmed that STZ induced oxidative stress in INS-1E cells (this study). Increasing the glucose concentration after STZ has been shown to protect against STZ cytotoxicity due to a neutralizing effect on oxidative stress [29]. This may explain the different mode of cell death at normal and elevated glucose levels in vitro, where high glucose could rescue beta cells from STZ-induced necrosis by increasing cellular NAD^+^ and making beta cells prone to oxidative stress-induced apoptosis.

Beta cell apoptosis could also be triggered as a result of increased ER stress. If ER stress reaches critically high levels, the ER stress response triggers caspase activation and BCL2-dependent programmed cell death in beta cells [12]. This is thought to play an important role in both beta cell lipo- and glucotoxicity [19]. STZ has been reported to induce ER stress leading to apoptosis in beta cells, hepatocytes and kidney cells [30–33]. Our previous work demonstrated that, under lipotoxic conditions, PPAG protected against ER stress-induced beta cell apoptosis by protecting the anti-apoptotic protein BCL2 from degradation whereas it did not affect expression and activation of pro-apoptotic proteins [5]. Lipotoxicity induced by high fat-containing western diets is considered a major contributor to the pathogenesis of type 2 diabetes. In the present study we confirmed that PPAG protects human islets from lipotoxicity.

Our present results show that PPAG can act as a cytoprotectant preventing beta cell death induced by various insults that are thought to participate in the pathogenesis and progression of type 2 diabetes, including oxidative stress, gluco- and lipotoxicity. The phytochemical protects both against BCL2-dependent apoptotic and BCL2-independent necrotic cell death mechanisms. A full understanding of the molecular mode of action of PPAG cytoprotection requires further investigation.

Our study also shows that PPAG does not act as an antioxidant nor can it protect against extensive DNA damage, the latter probably explaining why beta cells eventually succumbed following high-dose STZ injury. Nevertheless, our observations reinforce the conclusion that PPAG represents a “broad spectrum” beta cytoprotectant. This makes it an interesting potential drug for therapeutic or preventive application in (pre-)diabetes.

## References

[1] Meier JJ, Bonadonna RC. Role of Reduced β-Cell Mass Versus Impaired β-Cell Function in the Pathogenesis of Type 2 Diabetes. Diabetes Care. 2013; 36:S113–S119. 10.2337/dcS13-2008 23882035 PMC3920783

[2] Song I, Muller C, Louw J, Bouwens L. Regulating the Beta Cell Mass as a Strategy for Type-2 Diabetes Treatment. Curr Drug Targets. 2015; 16:516–524. 25654737 10.2174/1389450116666150204113928

[3] McCarty MF. Nutraceutical resources for diabetes prevention—an update. Med Hypotheses. 2005; 64:151–8. 15533633 10.1016/j.mehy.2004.03.036

[4] Muller CJ, Joubert E, Pheiffer C, Ghoor S, Sanderson M, Chellan N, et al Z-2-(ß-D-glucopyranosyloxy)-3-phenylpropenoic acid, an α-hydroxy acid from rooibos (Aspalathus linearis) with hypoglycemic activity. Mol Nutr Food Res. 2013; 57:2216–2222. 10.1002/mnfr.201300294 23943314

[5] Mathijs I, Da Cunha DA, Himpe E, Ladriere L, Chellan N, Roux CR, et al Phenylpropenoic acid glucoside augments pancreatic beta cell mass in high-fat diet-fed mice and protects beta cells from ER stress-induced apoptosis. Mol Nutr Food Res. 2014; 58:1980–1990. 10.1002/mnfr.201400211 25044754

[6] Joubert E, de Beer D, Malherbe CJ, Muller N, Bonnet SL, van der Westhuizen JH, et al Occurrence and sensory perception of Z-2-(ß-D-glucopyranosyloxy)-3-phenylpropenoic acid in rooibos (Aspalathus linearis). Food Chem. 2013; 136:1078–1085. 10.1016/j.foodchem.2012.09.014 23122165

[7] Marais C, Steenkamp JA, Ferreira D. Occurrence of phenylpyruvic acid in woody plants: biosynthetic significance and synthesis of an enolic glucoside derivative. J Chem Soc Perkin 1. 1996; 1:2915–2918.

[8] Rooman I, Bouwens L. Combined gastrin and epidermal growth factor treatment induces islet regeneration and restores normoglycaemia in C57Bl6/J mice treated with alloxan. Diabetologia. 2004; 47:259–265. 14666367 10.1007/s00125-003-1287-1

[9] Asfari M, Janjic D, Meda P, Li G, Halban PA, Wollheim CB. Establishment of 2-mercaptoethanol-dependent differentiated insulin-secreting cell lines. Endocrinology. 1992; 130:167–178. 1370150 10.1210/endo.130.1.1370150

[10] Lupi R, Dotta F, Marselli L, Del Guerra S, Masini M, Santangelo C, et al Prolonged exposure to free fatty acids has cytostatic and pro-apoptotic effects on human pancreatic islets: evidence that β-cell death is caspase mediated, partially dependent on ceramide pathway, and Bcl-2 regulated. Diabetes. 2002; 51:1437–1442. 11978640 10.2337/diabetes.51.5.1437

[11] Cnop M, Abdulkarim B, Bottu G, Cunha DA, Igoillo-Esteve M, Masini M, et al RNA sequencing identifies dysregulation of the human pancreatic islet transcriptome by the saturated fatty acid palmitate. Diabetes. 2014; 63:1978–93. 10.2337/db13-1383 24379348

[12] Cunha DA, Hekerman P, Ladrière L, Bazarra-Castro A, Ortis F, Wakeham MC, et al Initiation and execution of lipotoxic ER stress in pancreatic beta-cells. J Cell Sci. 2008; 121:2308–2318. 10.1242/jcs.026062 18559892 PMC3675788

[13] Cunha DA, Igoillo-Esteve M, Gurzov EN, Germano CM, Naamane N, Marhfour I, et al Death protein 5 and p53-upregulated modulator of apoptosis mediate the endoplasmic reticulum stress mitochondrial dialog triggering lipotoxic rodent and human ß-cell apoptosis. Diabetes. 2012; 61:2763–2775. 10.2337/db12-0123 22773666 PMC3478544

[14] Cnop M, Ladriere L, Hekerman P, Ortis F, Cardozo AK, Dogusan Z, et al Selective inhibition of eukaryotic translation initiation factor 2α dephosphorylation potentiates fatty acid-induced endoplasmic reticulum stress and causes pancreatic beta-cell dysfunction and apoptosis. J Biol Chem. 2007; 282:3989–3997. 17158450 10.1074/jbc.M607627200

[15] Eizirik DL, Pipeleers DG, Ling Z, Welsh N, Hellerström C, Andersson A. Major species differences between humans and rodents in the susceptibility to pancreatic beta-cell injury. Proc Natl Acad Sci U S A. 1994; 91:9253–9256. 7937750 10.1073/pnas.91.20.9253 PMC44790

[16] Wu J, Yan LY. Streptozotocin-induced type 1 diabetes in rodents as a model for studying mitochondrial mechanisms of diabetic β cell glucotoxicity. Diabetes Metab Syndr Obes. 2015; 8:181–188. 10.2147/DMSO.S82272 25897251 PMC4396517

[17] Hayashi K, Kojima R, Ito M. Strain differences in the diabetogenic activity of streptozotocin in mice. Biol Pharm Bull. 2006; 29:1110–1119. 16755002 10.1248/bpb.29.1110

[18] Vetere A, Choudhary A, Burns SM, Wagner BK. Targeting the pancreatic ß cell to treat diabetes. Nat Rev Drug Discov. 2014; 13:278–289. 10.1038/nrd4231 24525781

[19] Bensellam M, Laybutt DR, Jonas JC. The molecular mechanisms of pancreatic beta cell glucotoxicity: recent findings and future research directions. Mol Cell Endocrinol. 2012; 364:1–27. 10.1016/j.mce.2012.08.003 22885162

[20] Szkudelski T. The mechanism of alloxan and streptozotocin action in B cells of the rat pancreas. Physiol Res. 2001; 50:537–46. 11829314

[21] Lenzen S. The mechanisms of alloxan- and streptozotocin-induced diabetes. Diabetologia. 2008; 51:216–26. 18087688 10.1007/s00125-007-0886-7

[22] Morgan NG, Cable HC, Newcombe NR, Williams GT. Treatment of cultured pancreatic B-cells with streptozotocin induces cell death by apoptosis. Biosci Rep. 1994; 14:243–50. 7772717 10.1007/BF01209729

[23] Watanabe A, Nishijima K, Zhao S, Zhao Y, Tanaka Y, Takemoto H, et al Quantitative determination of apoptosis of pancreatic β-cells in a murine model of type 1 diabetes mellitus. J Nucl Med. 2012; 53:1585–91. 10.2967/jnumed.111.102459 22930815

[24] Zong WX, Ditsworth D, Bauer DE, Wang ZQ, Thompson SB. Alkylating DNA damage stimulates a regulated form of necrotic cell death. Genes Dev. 2004; 18:1272–1282. 15145826 10.1101/gad.1199904 PMC420353

[25] Lenzen S, Drinkgern J, Tiedge M. Low antioxidant enzyme gene expression in pancreatic islets compared with various other mouse tissues. Free Radic Bio Med. 1996; 20:463–6. 8720919 10.1016/0891-5849(96)02051-5

[26] Tiedge M, Lortz S, Drinkgern J, Lenzen S. Relation between antioxidant enzyme gene expression and antioxidative defense status of insulin-producing cells. Diabetes. 1997; 46:1733–42. 9356019 10.2337/diab.46.11.1733

[27] Lieberthal W, Menza SA, Levine JS. Graded ATP depletion can cause necrosis or apoptosis of cultured mouse proximal tubular cells. Am J Physiol. 1998; 274:F315–27. 9486226 10.1152/ajprenal.1998.274.2.F315

[28] Saito Y, Nishio K, Ogawa Y, Kimata J, Kinumi T, Yoshida Y, et al Turning point in apoptosis/necrosis induced by hydrogen peroxide. Free Radic Res. 2006; 40:619–30. 16753840 10.1080/10715760600632552

[29] Pipeleers D, Van De Winkel M. Pancreatic B cells possess defense mechanisms against cell-specific toxicity. Proc Natl Acad Sci U S A. 1986; 83:5267–5271. 2941762 10.1073/pnas.83.14.5267 PMC323932

[30] Ahn C, An BS, Jeung EB. Streptozotocin induces endoplasmic reticulum stress and apoptosis via disruption of calcium homeostasis in mouse pancreas. Mol Cell Endocrinol. 2015; 412:302–308. 10.1016/j.mce.2015.05.017 26003140

[31] Zhu M, Guo M, Fei L, Pan XQ, Liu QQ. 4-phenylbutyric acid attenuates endoplasmic reticulum stress-mediated pancreatic β-cell apoptosis in rats with streptozotocin-induced diabetes. Endocrine. 2014; 47:129–37. 10.1007/s12020-013-0132-7 24347242

[32] Afrin R, Arumugam S, Soetikno V, Thandavarayan RA, Pitchaimani V, Karuppagounder V, et al Curcumin ameliorates streptozotocin-induced liver damage through modulation of endoplasmic reticulum stress-mediated apoptosis in diabetic rats. Free Radic Res. 2015; 49:279–89. 10.3109/10715762.2014.999674 25536420 PMC4389763

[33] Uetake R, Sakurai T, Kamiyoshi A, Ichikawa-Shindo Y, Kawate H, Lesato Y, et al Adrenomedullin-RAMP2 system suppresses ER stress-induced tubule cell death and is involved in kidney protection. PLoS One. 2014; 5:e87667. 10.1371/journal.pone.0087667 PMC3914859 24505304

